# Influences on physical activity and screen time amongst postpartum women with heightened depressive symptoms: a qualitative study

**DOI:** 10.1186/s12884-021-03847-w

**Published:** 2021-05-15

**Authors:** Maria Apostolopoulos, Jill A. Hnatiuk, Jaimie-Lee Maple, Ellinor K. Olander, Leah Brennan, Paige van der Pligt, Megan Teychenne

**Affiliations:** 1 School of Exercise and Nutrition Sciences, Deakin University, Melbourne, Australia; 2 Institute for Physical Activity and Nutrition (IPAN), School of Exercise and Nutrition Sciences, Deakin University, Geelong, Australia; 3 Centre for Maternal and Child Health Research, School of Health Sciences, City, University of London, London, UK; 4 School of Psychology and Public Health, La Trobe University, Wodonga, Victoria Australia

**Keywords:** physical activity, sedentary behaviour, depression, mental health, postnatal, determinants, strategies

## Abstract

**Background:**

Postpartum women are at higher risk of depression compared to the general population. Despite the mental health benefits an active lifestyle can provide, postpartum women engage in low physical activity and high screen time. Very little research has investigated the social ecological (i.e. individual, social and physical environmental) influences on physical activity and screen time amongst postpartum women, particularly amongst those with depressive symptoms. Therefore, this study sought to examine the influences on physical activity and screen time amongst postpartum women with heightened depressive symptoms.

**Methods:**

20 mothers (3–9 months postpartum) participating in the *Mums on the Move* pilot randomised controlled trial who reported being insufficiently active and experiencing heightened depressive symptoms participated in semi-structured telephone interviews exploring their perceptions of the key influences on their physical activity and screen time across various levels of the social ecological model. Strategies for promoting physical activity and reducing screen time were explored with participants. Thematic analyses were undertaken to construct key themes from the qualitative data.

**Results:**

Findings showed that postpartum women with depressive symptoms reported individual (i.e. sleep quality, being housebound, single income), social (i.e. childcare, social support from partner and friends) and physical environmental (i.e. weather, safety in the local neighbourhood) influences on physical activity. Postpartum women reported individual (i.e. screen use out of habit and addiction, enjoyment) and social (i.e. positive role modelling, social isolation) influences on screen-time, but no key themes targeting the physical environmental influences were identified for screen time. Strategies suggested by women to increase physical activity included mother’s physical activity groups, home-based physical activity programs and awareness-raising. Strategies to reduce screen time included the use of screen time tracker apps, increasing social connections and awareness-raising.

**Conclusions:**

Amongst postpartum women with heightened depressive symptoms, influences on physical activity encompassed all constructs of the social ecological model. However, screen time was only perceived to be influenced by individual and social factors. Intervention strategies targeting predominantly individual and social factors may be particularly important for this high-risk group. These findings could assist in developing targeted physical activity and screen time interventions for this cohort.

**Supplementary Information:**

The online version contains supplementary material available at 10.1186/s12884-021-03847-w.

## Background

Research suggests that engaging in regular physical activity is associated with considerable long-term health benefits including reduced risk of cardiovascular disease, obesity, type 2 diabetes mellitus, depression and bone and joint diseases [1, 2]. Sedentary behaviours (i.e. sitting/reclining behaviours which often includes screen-time such as smartphone and computer-use) are also independent risk factors for premature morbidity and mortality [3] as well as adverse mental health outcomes including depression [4] and anxiety [5] in adults. Depression is one of the leading causes of morbidity and mortality globally [6, 7]. Postpartum women within the first year of childbirth are at heightened risk of depression with approximately 10–19 % experiencing postnatal depression worldwide [8].

It is generally well known that physical activity is an important contributor to both the physical and mental wellbeing of women in the postpartum period [9]. However, this cohort engage in low levels of physical activity and high levels of sedentary behaviour, in particular screen time [10, 11]. Being a new mother is a life stage transition which presents a new set of challenges in relation to physical activity participation and engagement in screen time, particularly for those experiencing heightened depressive symptoms. Although much evidence exists regarding the social ecological (e.g., individual, social and physical environmental) influences on physical activity and screen time amongst the general population, little is known about the factors influencing physical activity and screen time in postpartum women, particularly those with postnatal depressive symptoms.

Previous studies exploring the influences on physical activity amongst postpartum women have mostly identified intrapersonal factors including time constraints, lack of energy, outcome expectations and motivation and confidence to be active [12–15, 10] as well as social factors such as having exercise companions [10, 14, 15] and social support from family and friends to be active [10, 12, 13, 15]. Amongst this population group, physical environmental influences on physical activity were less commonly reported with weather the only factor identified [10, 12, 14]. However, no studies have identified the influences on physical activity amongst postpartum women experiencing postnatal depressive symptoms.

Limited research has explored the influences on sedentary behaviour in a number of other population groups including adults [16–19, 4] and adults with depression [20, 21] yet most of these studies investigated predominantly demographic (non-modifiable) factors (e.g., age, gender, employment status). A small body of literature has suggested that social factors such as social cohesion, social network size and social support may influence sedentary behaviour in adult population groups [20, 17, 19]. Whilst physical environmental level influences such as neighbourhood walkability showed mixed associations with sedentary behaviour amongst adults [16], weather was found to be a key factor influencing sedentary behaviour amongst women experiencing depressive symptoms [22]. To date no studies have examined the influences on sedentary behaviour in postpartum women with or without depressive symptoms.

In order to increase engagement in physical activity and reduce the prevalence of sedentary behaviour (in particular screen time) amongst this cohort, it is essential to explore the key influences on these behaviours. Therefore, the aim of this study was to investigate the perceived influences on physical activity and screen time amongst postpartum women experiencing heightened depressive symptoms. Furthermore, this study explored potential strategies that women believed could assist them to increase participation in physical activity and reduce engagement in screen time during the postpartum period. Given little is known regarding the influences on physical activity and screen time amongst this population group, qualitative methods are particularly useful as they provide in-depth understanding and elicit rich insights in areas of research that are poorly understood [23].

## Methods

### Study design

A phenomenological approach [24] was employed in the one-on-one telephone interviews conducted with a subset of postpartum women experiencing depressive symptoms who were participating in the *Mums on the Move* pilot randomised controlled trial (Australian New-Zealand Clinical Trial Registry: ACTRN12618001453279. Registered 29th August 2018. Deakin University Ethics Project Number: 2018 − 139). The focus of a phenomenological approach is on interpreting meaning of an experience and creating themes that capture the lived experiences of individuals or groups [24]. Ethics approval to conduct the research was obtained from the Deakin University Human Research Ethics Committee (DU-HREC-2018-139). To strengthen methodological rigour and transparency, reporting follows the Standards for Reporting Qualitative Research (SRQR) [25].

### Recruitment

Participants were recruited from the *Mums on the Move* cohort, which consisted of 62 postpartum women residing in metropolitan Melbourne [26]. Women were eligible for inclusion in the larger study if they were 3–9 months postpartum, classified as ‘at-risk’ of postnatal depression (i.e. those who scored ≥ 10 on the Edinburgh postnatal depression scale (EPDS)) [27], which is a validated tool for detecting the likelihood of depression [28], not currently taking anti-depressant medication, insufficiently active (i.e. not meeting the recommended 150 min/week of moderate-intensity physical activity) and not currently owning exercise equipment (i.e. treadmill or stationary bike). These inclusion criteria were specified for the purposes of the larger RCT (which tested the efficacy of a home-based intervention designed to overcome barriers to physical activity amongst postpartum women experiencing heightened depressive symptoms) and described in further detail elsewhere [26]. A total of 1,614 women completed the online screening survey and of those 1,352 were excluded due to not meeting the eligibility criteria. A total of 262 women were eligible for inclusion and provided the research team with their contact details. Eligible participants were required to provide written consent and to obtain medical clearance from their GP in order to be recruited to the study. A total of 68 women returned their consent form and GP clearance letter however, six women could not be included in the study as the maximum number of participants the budget would allow was exceeded. These women were thanked for their interest in the study and notified via email that they could not be included for this reason. Thus, the final sample for the larger study consisted of the first 62 women who met the study requirements. Of the 62 women, 41 women indicated they would be interested in participating in a qualitative sub-study and of these the first 20 women were recruited into the current sub-study. A total of 20 participants was theoretically determined to enable data saturation [29] and during the data analysis stage this was then confirmed to be sufficient.

*Mums on the Move* participants were recruited over a 12-week period throughout July-October 2018 using paid and unpaid social media advertising (i.e. Facebook and Instagram) as well as information flyers distributed to local Maternal and Child Health Centres (MCHC). Interested participants completed an online screening questionnaire to determine eligibility for the study. Eligible participants were required to provide informed consent (via an online tick-box), GP clearance and indicate their interest in participating in a qualitative sub-study prior to enrolment in the program. Participants who consented to the sub-study were contacted via email by the researcher to arrange a time for the one-on-one telephone interview. Telephone interviews were chosen as the data collection method as it allows participants to disclose sensitive information (i.e., struggles experienced during the postpartum period) in a more comfortable environment [30]. Logistically this is easier given postpartum women are likely to face challenges leaving the home [26] so this method would help study recruitment. However, telephone interviews may make it difficult to establish interviewer-interviewee connection (compared with face-to-face methods) which could limit the depth of responses [30].

### Interview procedures

A semi-structured interview schedule was developed and refined by the research team. One-on-one qualitative interviews were conducted with participants over the telephone by an experienced research assistant prior to randomisation. The interviews lasted for approximately 20 min. Telephone interviews were audio-recorded, and participants received a $25 store gift voucher as compensation for their time.

### Interview schedule

The semi-structured interview schedule was guided by the social ecological model [31] and included open-ended questions with prompts. These questions assessed the individual, social and physical environmental level influences on physical activity and screen time, as well as ideas on potential strategies that may be effective in assisting postpartum women to increase physical activity and reduce screen time (see Supplementary Table 1).

### Influences on physical activity

Participants were asked about the key individual, social and physical environmental level influences that either facilitated or inhibited their participation in leisure-time physical activity. Questions included: ‘Are there any individual/personal factors that prevent you from being physically active?’ (prompts included lack of time); ‘Are there any social factors that enable/help you to be active?’ (prompts included having someone to be active with/ social support); ‘Are there any physical environmental factors that prevent you from being physically active/exercising?’ (prompts included access to facilities).

### Influences on screen time

Participants were asked about the amount of time spent engaged in recreational screen time per day, the type of screen behaviour engaged in most (i.e. smartphone, tablets, computer or television viewing) as well as key reasons for engaging in this behaviour (i.e. entertainment, boredom, habit). Barriers in relation to recreational screen time use were explored with participants. Questions included: ‘If you had to have a recreational screen-free week, what would be the key individual factors that would prevent you from switching off [most commonly used screen behaviour]?’ (prompts included boredom, addiction to screen behaviours). Screen time facilitators were explored with questions such as ‘Are there any social factors that enable/ help you to switch off [most commonly used screen behaviour] during your leisure-time?’ (prompts included friends/family discourage, positive role modelling for children). Physical environmental facilitators were also explored by asking ‘Are there any environmental factors that enable/help you to switch off [most commonly reported screen behaviour]?’ (prompts included cost of Wi-Fi and Netflix).

### Strategies to increase physical activity and reduce screen time

Suggestions for potential strategies to increase leisure-time physical activity and reduce recreational screen time were prompted with questions such as ‘What one strategy do you think would be most effective/important to help increase physical activity amongst postpartum women?’ and ‘Can you think of a strategy that would be effective/important to help reduce recreational screen time amongst postpartum women?’.

### Data Analysis

Descriptive statistics on participant demographic data were summarised using SPSS software (version 25). Interviews were conducted in 2018 and were audio-recorded then later transcribed in full. The interviewer/ researcher (MA) took detailed notes during these interviews with key points and themes summarised at the end of each interview. In order to explore various possible interpretations of the data, a random subset of two (10 %) interview transcripts were independently coded by two authors (MA and MT) at the commencement of data analysis [32]. Both authors met and discussed their interpretations of the data with no major discrepancies in interpretation identified. Member checking was not used in the analysis [33]. Thematic analysis following an inductive approach was utilised [34, 35]. Even though the social ecological model was used as a guide in the analysis, the approach was considered inductive because it was data-driven rather than having any parameters restricting the analysis [35]. NVivo software for qualitative data analysis was used to organise the data. Thematic analysis was performed following phases outlined by Braun and Clarke [36]. First, familiarization with the data took place whereby the interviewer (MA) repeatedly read the interview transcripts (Phase 1) and then initial sub-categories (i.e. nap and feeding times) were generated from the data within the NVivo software (Phase 2). The next step involved searching for themes and creating major categories by combining similar sub-categories (i.e. baby routines) (Phase 3). Following this, the themes created (i.e. the influence of being housebound) and the thematic map were reviewed (Phase 4). Final definitions and names (i.e. being housebound) were then generated for each theme constructed from the data (Phase 5). The final stage (Phase 6) involved the analysis and write-up of results. In order to present key themes, written participant quotes are presented with these anonymised using an assigned pseudonym and actual age for each participant.

### Researcher reflexivity

MA (BExSportSci (Hons)) was the project manager of the *Mums on the Move* pilot randomised controlled trial and undertook interviews with participants in the qualitative sub-study. She undertook data analysis and has previous experience in qualitative data analysis in the area of physical activity. MT (PhD) is a behavioural epidemiologist conducting research in the area of physical activity and mental health. MT was the *Mums on the Move* project lead and was responsible for the design/ development of the intervention. All remaining authors were not involved in the qualitative interviews and analysis of the data however, they were given the opportunity to review themes and suggest different interpretations of the data during the final stage of the thematic analysis (Phase 6). Postpartum women (participants) were not known to MA, and both authors (MA and MT) were involved in cross-checking the data to ensure consistency.

## Results

**Fig. 1:**
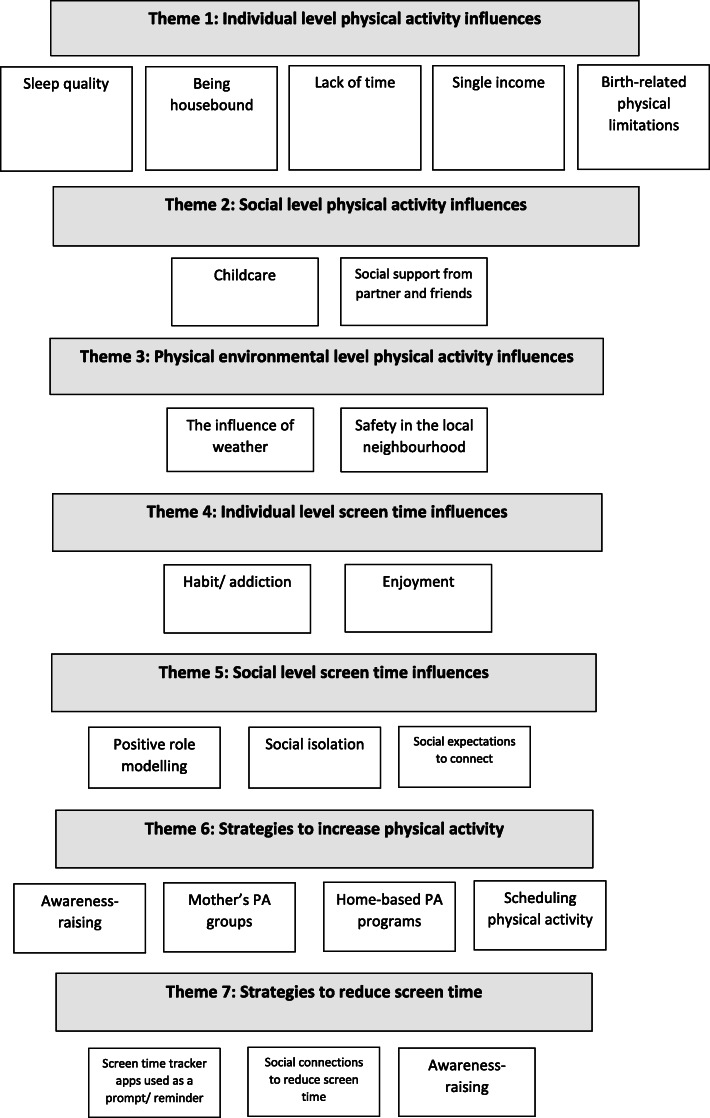
Themes and sub-themes constructed from qualitative data

This sample (*n* = 20) of postpartum women were between 24 and 39 years old and were 12 to 36 weeks postpartum. The sample included both primiparous (55 %) and multiparous (45 %) women. The participants were from various nationalities, but most were Australian (60 %) or English (25 %). The majority of participants were married/ de facto (95 %) and most (75 %) had a University degree/ post-graduate qualification. In regard to employment, most women (70 %) were classified as not working/ household duties but of those who had resumed employment (30 %) the average time spent in paid or voluntary work was 9.16 h per week (Fig. 1).

### Influences on physical activity

Key themes encompassed the individual (i.e. sleep quality, being housebound, lack of time, single income, and birth-related physical limitations), social (i.e. childcare, social support from partner and friends) and physical environmental (i.e. weather, safety in the local neighbourhood) constructs of the social ecological model.

### Individual

#### Sleep quality

A distinct theme was the influence of sleep deprivation on postpartum physical activity with most women reporting that a lack of energy due to broken sleep was a significant barrier to physical activity participation.

*“Having such broken sleep. It’s kind of like I’m lucky to get five hours of broken sleep. The thought of then trying to exercise during the day, I’m just too tired”* (Natalie, 37).

#### Being housebound

Most women felt ‘housebound’ which prevented them from being active due to baby routines and unsettled babies. Many women mentioned that infant nap and feeding routines impacted on their ability to commit to structured exercise classes, social walking groups and mums and bubs classes offered within the community.

*“I mean it’s so unpredictable. There are some weeks when you can say I’ve got this sorted. Yes, I can meet you for a walk, 10:30 every morning and then by the following week, you’re teething or– something has happened, then everything changes. I think having a routine that’s predictable is impossible with a new baby. It makes it difficult to commit to anything”* (Linda, 39).

Additionally, some women cited the perceived effort of leaving the house with their baby and children as a factor that often prevented them from being active.

*“Yeah, so, sort of wanting to go and do stuff and then thinking, I’ve got to get someone over to look after the kids or just go out for a bit walk all right, now I’ve got to find the shoes, got to get this one ready, this one needs food, this one needs a drink, you know, there’s lots of like leaving the house kind of things. Then you just think, it’s too hard”* (Anna, 35).

#### Lack of time

Lack of time was commonly reported as a barrier to physical activity. The perceived causes of lack of time tended to focus on parenting demands, and prioritising the needs of the baby and household chores.

*“Her routine, as much as a routine that you can have with a six-month-old. Her routine and needs and time schedule, completely trump every little aspect in my life”* (Danielle, 38).

#### Single Income

The cost associated with physical activity was a barrier to being active for about a quarter of women who were on maternity leave and now living off a single income. The cost of gym memberships, mums and bubs classes and exercise equipment were commonly mentioned as a barrier.

*“So being on maternity leave, you know, yet again with four kids, you know, where money is tight, which means that I’ve been I just don’t have the disposable income to join a gym”* (Jade, 34).

#### Birth-related physical limitations

A few women cited birth-related physical limitations such as pain related with a caesarean birth and abdominal muscle separation, as a factor that prevented them from being physically active as they wanted to ensure their bodies could cope with the physical demands of being active and avoid further damage or injuries.

*“I had an emergency caesarean and I’ve had a fair bit of pain since, in my lower abdomen. I’ve seen a physio about that, but that’s also made me a little bit nervous. So right now, I’ve had no pelvic floor issues but it’s more just that it’s still really tender where they cut me open”* (Megan, 33).

*“I’ve got a separation in my stomach muscles and I just make sure I’m not doing myself more damage because that’s happened in the past when I have done too much and my stomach muscles haven’t been strong enough to cope and you end up with an injury”* (Sandra, 24).

### Social

#### Childcare

One of the most common themes identified was the influence of childcare on being active in the postpartum period. Most women suggested that a lack of childcare or babysitting options was a factor that significantly inhibited their ability to be active. Key reasons included family living overseas resulting in limited childcare options.

*“Well, I guess, the main one for me would be childcare because I don’t have family living here in Australia”* (Sarah, 33).

However, one woman mentioned that having grandparents as a childcare option enabled her to be physically active.

*“I’ve got grandparents, you know, so the kids have their grandparents around that are happy to help which makes it easier for me to be active”* (Jade, 34).

#### Social support from partner and friends

Most of the women described having support and encouragement from their partner as imperative in enabling them to be active. Further, some mothers stated that they were active with their partners and children through activities such as walking and bike rides.

*“Yeah, my husband will say let’s go for a walk and pick up my daughter from childcare all together”* (Anna, 35).

The presence of social networks, including mother’s groups were viewed as both an opportunity to socialize with other mums and be active. More than half the women suggested that they often walked with mothers from their parents’ group, and a couple of women mentioned using social media forums to connect with mums in the neighbourhood to organise walks.

*“So, at the moment it’s the mum’s group encouraging each other to go out for walks when the weather is nice so that’s been a facilitator”* (Jacqui, 34).

### Physical environmental

#### The influence of weather

A distinct theme that emerged from the one-on-one interviews was the influence of weather on physical activity. Most women suggested that cold weather and rain would be a barrier preventing them from being active outdoors or venturing out with their baby.

*“I don’t want to put a newborn baby in the pram with a chance that it’s going to rain which has been pretty much the story every day since he has been born. Definitely having a winter baby has kept me inside more than I would have been”* (Jennifer, 29).

Conversely, some women mentioned that pleasant weather increased their motivation to be active and had mood-enhancing effects which were seen as conducive to physical activity.

*“Well, for me, one thing, is the weather. Certainly, my mood is better when the weather is better and I try to sneak out for 20 minutes and go for a run on those days”* (Isabella, 32).

#### Safety in the local neighbourhood

Safety concerns in the local neighbourhood were described as a key influence on physical activity by this cohort. Most women suggested that they would not engage in physical activity in the dark due to safety concerns regardless of how safe they perceived their neighbourhood to be.

*“I mean I wouldn’t walk– I live in a pretty– a relatively safe area. But I still wouldn’t go for a walk or run on my own in the dark”* (Olivia, 31).

### Influences on screen time

Recreational screen-time use of women, which included time spent using smartphones, tablets, computers and television viewing for leisure or recreational purposes, were explored. The most commonly reported form of recreational screen time in this cohort of women were smartphones followed by television viewing. Key reasons for engaging in smartphone use were to scroll through and engage with social media platforms (i.e. Facebook, Instagram), to connect with others (i.e. text messaging, group chats), as an information source (i.e. parenting websites), out of boredom, procrastination to avoid household chores and as a mindless distraction.

Key themes constructed to determine the influences on screen time encompassed the individual (i.e. habit/addiction and enjoyment) and social (i.e. positive role modelling, social isolation, social expectations to connect) constructs of the social ecological model. No themes targeting physical environmental level influences on screen time were identified.

### Individual

#### Habit/addiction

Most mothers spoke at length about the habit or addiction they had developed to screen time, in particular smartphone use. ‘Mindless scrolling’ through social media platforms was mentioned by mothers as a strategy they used to switch off and overcome feelings of boredom.

*“That like time passing addiction of what should I do right now. I’ve got a couple of minutes, I’ll check Facebook. It’s definitely there”* (Claudia, 34).

*“I don’t feel like I’ve got the ability to concentrate. So, instead of being on my phone, I could read a book. I’d read the same page over and over because I just don’t take it in. Whereas, being on my phone is a bit mindless. You don’t have to think too much”* (Mia, 38).

#### Enjoyment

A few women described how their enjoyment of screen time (i.e. smartphones, television viewing) would be a factor that would make it difficult for them to switch off screens.

*“Um, I guess I enjoy it so it would be difficult to switch it off because I look forward to couch time in front of the TV when the kids are in bed”* (Sandra, 24).

### Social

#### Positive role modelling

Demonstrating positive role modelling to children in relation to screen time behaviours, particularly smartphone use, was suggested by most women as a motivation for limiting their own screen time. Most mothers described how they were conscious of screen time use when in the presence of their children as they wanted to be a positive role model and ensure that their child felt valued, through being ‘present’ as a parent.

*“When I’m with the baby, I really try not to use it and I’m really conscious about it because she’s watching me and she’s looking for my eye contact and I’ve noticed if I have picked up my phone, she’s looking at me and I’m looking at my phone and I just think that that’s awful”* (Isabella, 32).

#### Social isolation

Feelings of social isolation and loneliness were described by most women as an influence on screen time. Some women mentioned that smartphone use was their link to the outside world when feeling isolated at home with their baby.

*“I think especially during the day when I’m home alone it’s that way of feeling some sort of connection that you’re still part of the world rather than being quite isolated. I live about 40 minutes away from my nearest friends that had a baby. It’s sort of that link to your old life and old connections”* (Nadine, 31).

Conversely, some women cited that having face-to-face connection with others or being occupied with other tasks would enable them to switch off their screens.

*“If we’re going for a walk or something or if I’m just playing with my baby and he’s happy then I’m happy to engage and don’t need the smartphone during those times”* (Jacqui, 32).

#### Social expectations to connect

The social expectation to connect via screen time (particularly smartphones) was viewed as a barrier preventing postpartum women from switching off screens in their leisure time. A number of women stated they used smartphones to connect with others (i.e. via text messaging, group chats and social media forums such as Facebook), share photos of their baby, to arrange social catch ups and to feel connected to the outside world.

*“I guess I would feel disconnected from my friends and family……we organise catch ups and things all on social media so that’s how we organise”* (Julie, 34).

*“So, I spend a lot of time on Facebook or WhatsApp, sending photos of my daughter or just catching up. But also missing out on catching up with people, because that’s how as a big group how we all communicate”* (Lauren, 31).

### Potential strategies to increase physical activity amongst postpartum women

A number of different strategies to increase physical activity were mentioned by the participants and are summarised below.

#### Strategy 1: Awareness-raising

A strategy that was frequently suggested to increase physical activity was educating postpartum women on the physical health benefits associated with postpartum physical activity as well as providing guidance on when and how to resume physical activity after giving birth. Many women felt they received little information from their GP or maternal child health nurse about postnatal recovery including recommendations of appropriate exercises that would assist in the recovery from childbirth.

*“I had a C-section and I wasn’t given any advice, support or any recommendations of what I should or shouldn’t be doing. I think that’s a big factor that people just, I don’t know, I just assumed especially having a C-section that I would have been given exercises to do and things like that when I first started”* (Lauren, 31).

In addition, some women suggested the provision of information regarding available exercise programs and facilities within the local neighbourhood would be an effective strategy to increase postpartum physical activity.

*“This program happens on an oval that I can walk to. I didn’t even know it was there until a friend of mine mentioned it to me. So, it’s being more aware of what may be on offer”* (Nadine, 31).

#### Strategy 2: Mother’s physical activity groups

Engaging more social support for physical activity from other postpartum women was the one of the most frequently suggested strategies to increase physical activity amongst participants in this study. Most women stated that being involved in a mother’s exercise/ walking group would provide the necessary social support and motivation to be active.

*“I think a walking group for new mums would be great. I think that’s definitely a place to start, because you’re wanting to build up those social connections, so I think, for me, that would be a great strategy”* (Jane, 31).

#### Strategy 3: Home-based physical activity programs

Several women suggested that home-based physical activity programs or access to exercise equipment within the home could be an effective strategy to increase physical activity given that most postpartum women are relatively housebound due to a lack of childcare and strict baby routines (i.e. nap and feeding times).

*“Yes, so something in the house that is ready to use. Absolutely I would use it because it’s there and you know you’re not going anywhere. You’re not leaving the house and basically, it’s the only way I can see that it’s quite an easy way of exercising for new mums”* (Jacqui, 32).

#### Strategy 4: Scheduling physical activity

A small proportion of women suggested that scheduling physical activity at set times throughout the week like an appointment would assist them in establishing routines and keeping them accountable to their activity regime.

*“For me, I think I know what works best for me is when I have an appointment, I can’t let anyone down. So, I have to sort of make that appointment with myself”* (Jennifer, 29).

### Potential strategies to reduce screen time amongst postpartum women

#### Strategy 1: Screen-time tracker apps used as a prompt/reminder

Employing the use of screen time tracker apps as a prompt or reminder of how much time is spent using smartphones was a key strategy suggested to reduce screen time in postpartum women. Most women believed that this strategy would provide the necessary motivation to switch off.

*“I try to have that app that tracks your screen time and then I’ll be like “Oh my God, I’ve used it too much”* (Jennifer, 29).

#### Strategy 2: Social connections to reduce screen-time

Many women believed that increasing social connections and having more face-to-face contact with others would be an effective strategy to reduce recreational screen time in postpartum women who are relatively isolated.

*“I think that social connections are really important, because I think that’s when screen time comes into things when you’re feeling isolated or alone. And so, if you’re able to encourage more group activities or social stuff, whether it’s partnering up with someone that would be a really good way to kind of reduce that screen time”* (Nadine, 31).

#### Strategy 3: Awareness-raising

Another commonly mentioned strategy for reducing screen-time was awareness-raising to educate postpartum women on the negative effects of screen time. Some mothers suggested that this education could be provided by health professionals and guest speakers.

*“You could maybe have some professionals coming out or a few guest speakers discussing that side of it. One side discussing, promoting physical activity and then the other side could be more about sitting less, using less screen time and smartphones”* (Mia, 38).

## Discussion

This is the first study to describe the social-ecological influences on physical activity and screen time amongst postnatal women experiencing heightened depressive symptoms. A key finding amongst this cohort was that the influences on physical activity and screen time primarily encompassed the individual and social levels of the ecological model.

### Influences on physical activity

Postpartum women in the current study commonly expressed how the influence of sleep deprivation and a lack of energy had a significant impact on their participation in physical activity. This finding is consistent with previous studies [12, 14] that reported lack of sleep as a key barrier to postpartum physical activity. In addition, other individual level influences on physical activity identified in the current study included ‘being housebound’ due to baby routines and a ‘lack of time’. These barriers were often the result of women prioritising the needs of their baby (i.e. nap and feeding times) over their own health and wellbeing. Time constraints is a key barrier to physical activity reported by postpartum women in previous studies [12–15] and often explained by parenting demands and domestic duties which are consistent with the findings of this study. This highlights the importance of developing physical activity interventions that have a strong focus on the time constraints and new demands experienced by women in the postpartum period. A noteworthy theme that emerged in the current study was the cost associated with physical activity as many women were adjusting to now being a single-income family while on maternity leave. This is an important finding as previous research has not identified this as a significant influence on postpartum physical activity and highlights the importance of low cost or free activities for this cohort.

The influence of social support from partners, family and friends was identified as a facilitator to postpartum physical activity in this study. Most women reported that encouragement from partners and the presence of social support networks including mother’s groups provided the necessary support to be active. This finding is supported by previous studies [10, 12, 13, 15] that found family and friend support (i.e. encouragement, babysitting) was conducive to postpartum physical activity. The influence of weather as well as safety within the local neighbourhood were identified as barriers by participants. This finding is not surprising as this study was conducted in winter which generally presents a new set of challenges in relation to physical activity participation (e.g., cold weather, rain). Previous studies [10, 12, 14] have also found that weather was a factor influencing postpartum physical activity. However, safety concerns in the local neighbourhood were not considered a key factor in those studies. These findings highlight the importance of developing convenient, flexible, cost-effective, and safe physical activity interventions for postpartum women that incorporate social support strategies such as walking groups delivered at flexible times or home-based programs that include elements of social support (e.g. partner education, social media strategies). Thus, future interventions targeting these key individual, social and physical environmental level influences on postpartum physical activity are needed.

### Influences on screen time

Postpartum women in the current study commonly expressed how screen time, in particular smartphone use, had become somewhat of a habit or addiction. This finding is consistent with previous studies [37, 38] that reported women were more likely to develop habitual or addictive smartphone behaviours in comparison to men. ‘Mindless scrolling’ was the term used by mothers to describe the behaviour of scrolling through social media platforms to ‘switch off’ and ease feelings of boredom. A similar theme was identified in previous research amongst women with depressive symptoms [22] whereby they used television viewing as a tool to ‘switch off’, divert negative thoughts and ease depressive symptoms. This finding emphasises that we need to be mindful of the motive behind screen time use amongst postpartum women and adapt our screen time intervention strategies accordingly to suit the needs of this cohort. This could involve incorporating mindfulness meditation strategies within screen time interventions designed for postpartum women to assist them in ‘switching off’. This approach has been useful in assisting individuals to cope with the stresses of daily life as well as benefiting those suffering from depression [39].

Another theme that emerged from the current study was the influence of demonstrating positive role modelling to children in relation to screen time behaviours. Many women suggested that they wanted their child to feel valued and this made them more conscious of their screen time behaviours when in the presence of their children. Literature suggests that reducing parental screen time can result in a reduction in child screen time [40]. Together, these findings highlight how encouraging positive parental role-modelling might be used as a stealth-based approach to reduce screen-time amongst postpartum women, particularly those experiencing depressive symptoms. Another key social level influence on screen time identified in the current study was social isolation and loneliness which were perceived as barriers to limiting screen time amongst this cohort. Many mothers reported that they felt isolated at home with their baby and that using smartphones was their link to the outside world. Therefore, future interventions must incorporate social support strategies (e.g., having an active buddy) [41] to reduce engagement in screen time and decrease postnatal depressive symptoms [42] amongst mothers.

### Physical activity and screen time strategies

Some strategies proposed by women to increase physical activity and reduce screen time were distinct. Home-based physical activity programs and scheduling physical activity at set times throughout the week were suggested by women as strategies for increasing postpartum physical activity specifically, which may help overcome the key barriers of ‘lack of time’ and ‘being housebound’. Women also suggested using screen time tracker apps on smartphones as a ‘reminder’ of how much time is spent on smartphones, which may ‘prompt’ women to specifically reduce screen time. Although limited research has investigated the effectiveness of screen time tracker apps in changing behaviours, one study using a qualitative methodology found that personal tracking of device use was potentially useful in reducing screen use amongst students [43].

Despite the influences on physical activity and screen time being completely distinct from each other in this study, a number of potential intervention strategies to both increase physical activity and reduce recreational screen time were proposed by women. A common theme that emerged was the emphasis on awareness-raising and education on the health benefits associated with physical activity and providing guidance on resuming physical activity post-childbirth. Similarly, many women believed education on the negative effects of screen time via health professionals and guest speakers would help reduce recreational screen time amongst mothers. Awareness-raising and education has been previously suggested to be a potential strategy for promoting physical activity and/or reductions in sedentary behaviour [22]. However, whilst important, it is generally only effective when used in conjunction with other strategies to change behaviour [44].

The importance of building social connections and engaging social support from other postpartum women was another common strategy suggested for increasing postpartum physical activity and reducing engagement in screen time. Most women suggested that being involved in an exercise group with other mothers would provide the necessary support to be active. Furthermore, increasing social connections and face-to-face interaction with others was suggested to reduce social isolation as this tended to result in more screen time amongst this cohort. Previous efficacious interventions conducted with postpartum women reporting depressive symptoms [45, 46] incorporated social support strategies (i.e. pram-walking groups, support sessions) to increase physical activity amongst this cohort. However, it is important to consider that postnatal depression is associated with social withdrawal [47, 48] and hence it is likely that depressed populations will be less inclined to engage in parent or exercise groups. Thus, it is imperative to develop programs that involve family members and friends to educate them on how to best support the mother in not only increasing physical activity and reducing screen time, but also for mental health.

### Study Limitations/ Strengths

Several methodological issues need to be considered when interpreting the findings presented in this study. This study sample primarily comprised postpartum women who were predominantly Australian, married/ de facto, tertiary educated and living in metropolitan Melbourne. Thus, the results may not be applicable to all postpartum women (i.e. ethnically diverse, lower level of education, residing in rural areas). However, given this was an exploratory study aiming to provide in-depth explanations, applicability was not the main objective [49]. It is important to note that single postpartum women included in the sample may face distinct or additional barriers to increasing physical activity and reducing screen time compared to those experienced by postpartum women who were married or in a de facto relationship. Although it was beyond the scope of this study to compare results between primiparous and multiparous women, it is likely that barriers may also differ for these women and as such future research must explore these differences. In addition, women were not all similar in postpartum stage (i.e. some were 3-months while others were 9-months postpartum) which may have influenced results given that the physical activity/ screen time influences identified could vary based on the age of the baby, their routines and development. However, this could also be considered a strength in that this study captured the views of women across a large proportion of time within the first year after childbirth. Measures taken to ensure trustworthiness of the data included keeping detailed records or an audit trail which ensured the steps involved in the research were transparent [32]. In addition, the semi-structured interview audio recordings were re-visited on numerous occasions to ensure that the experiences of postpartum women were accurately represented in the themes constructed from the data [49]. The inclusion of data rich descriptions of participants thoughts and experiences in relation to postpartum physical activity and screen time influences enhanced the credibility of the study findings [49]. A strength of the study was the qualitative design, which provided rich insights into the topic not possible from quantitative methods. This qualitative study is also the first to have explored the key influences on physical activity and screen time amongst this high-risk target group of postpartum women experiencing depressive symptoms.

## Conclusions

Findings from the current study have important implications for physical activity and screen time intervention research amongst postpartum women. Strategies to increase physical activity and reduce screen time could involve providing postpartum women with education (i.e. from health professionals including their GP or maternal child health nurse), resources (i.e. exercise equipment within the home) and supporting social support (i.e. developing supportive social systems through mother’s physical activity groups in the local neighbourhood) to assist with positive behaviour change. Intervention strategies that don’t target behaviour change directly are unlikely to be effective in increasing physical activity and reducing screen time in postpartum women. These findings could enable the development of targeted interventions that are tailored to the needs of this unique cohort, which is crucial to the success and sustainability of future programs. Future interventions must target key individual and social level influences on screen time identified in this study to ensure program success. These findings are important as they contribute to the lack of existing literature in relation to the influences on screen time amongst postpartum women, particularly those experiencing heightened depressive symptoms.

## Supplementary Information

**Additional file 1: **

## Data Availability

The transcripts/datasets analysed during the current study are not publicly available due to ethical restrictions (participants have not consented to the use of their data for purposes other than those for which they originally consented). Should a researcher request the data for a particular purpose, an ethically compliant dataset may be made available via the senior author upon approval by the Deakin University Human Research Ethics Committee. Requests can be emailed to: research-ethics@deakin.edu.au.

## Abbreviations

EPDS: Edinburgh Postnatal Depression Scale
GP: General Practitioner
MCHC: Maternal and Child Health Centres
SRQR: Standards for Reporting Qualitative Research
