# Supplementary material for: Influences on physical activity and screen time amongst postpartum women with heightened depressive symptoms: a qualitative study
Source: BMC Pregnancy Childbirth. 2021 May 15;21:376. doi: 10.1186/s12884-021-03847-w (PMC8126115; doi:10.1186/s12884-021-03847-w)
Supplement: Supplementary file 1 — Additional file 1: [file 12884_2021_3847_MOESM1_ESM.docx]

**Supplementary table 1.** Qualitative sub-study interview schedule

| ***Physical Activity Barriers***  1) What kinds of factors currently prevent you from being physically active/exercising?  - Depending on answer prompt with:  2) Are there any individual/personal factors that prevent you from being physically active/exercising? (Prompts: feeling self-conscious/ weight, lack of time, lack of motivation, lack of confidence, lacking energy/ feeling tired, low mood/feeling down)  3) Are there any social factors that prevent you from being physically active/exercising? (Prompts: lack of childcare, parental responsibilities (feeding, unsettled baby), lack of social support to be active, did others discourage you from being active etc.)  4) Are there any physical environmental factors that prevented you prevent you from being physically active/exercising? (Prompts: weather, access to facilities, neighbourhood safety/ attractiveness). |
| --- |
| ***Physical Activity Facilitators***  1) What factors enable or help you to be physically active/exercise?  - Depending on answer prompt with:  2) Are there any individual factors that enable/help you to be active? (Prompts: Prior history/ enjoyment of activity, more time, confidence to be active)  3) Are there any social factors that enable/help you to be active? (Prompts: having someone to be active with/ social support, others encouraging you to be active, childcare)  4) Are there any physical environmental influences that enable/help you to be active? (Prompts: parks/ walking tracks nearby, access to gyms/ fitness centres etc.) |
| ***Screen time Barriers***  *Now we are going to talk about your recreational screen time. This includes time that you spend using smartphones, tablets (e.g. iPad) computers and TV for leisure or recreational purposes (so not for work).*  Of these, which screen behaviour would you spend most time engaged in (smartphone, tablets, computer or TV)? _________________________. How much time per day would you spend engaged in [enter which behaviour they answered here]? ______________. What is the key reason for engaging in [enter which behaviour they answered here]? _______________________________. (Prompts: entertainment, social boredom, addiction, feeling like they are missing out, education/ learning including parenting websites) |
| 1) If you had to have a recreational screen-free week, what would be the key factors that would prevent you (or make it difficult to) switch off [enter that screen behaviour here]?  - Depending on answer prompt with:  2) Are there any individual factors that prevent you from switching off [enter that screen behaviour here] during your leisure-time? (Prompts: bored, feeling addicted, enjoyment, lack self-efficacy to switch off, lack motivation, too tired, fear of missing out, require it for education/learning)  3) Are there any social factors that prevent you from switching off [enter that screen behaviour here] during your leisure-time? (Prompts: the way you socially connect with people, friends/family expectations/encouragement, social norm, mummy blogs – connecting with mothers)  4) Are there any physical environmental factors that prevent you from switching off [enter that screen behaviour here] during your leisure-time? (Prompts: Easy access, weather) |
| ***Screen Time Facilitators***  1) What currently enables/helps you to switch off [enter that screen behaviour here] during your leisure-time?  - Depending on answer prompt with:  2) Are there any individual factors that enable/help you to switch off [enter that screen behaviour here] during your leisure-time? (Prompts: knowledge of health risks due to using screens/high SB, Lack of time, lack of enjoyment, high motivation)  3) Are there any social factors that enable/help you to switch off [enter that screen behaviour here] during your leisure-time? (Prompts: friends/family discourage, role modelling/trying to set good example for children, friends/family don’t connect that way)  4) Are there any environmental factors that enable/help you to switch off [enter that screen behaviour here] during your leisure-time? (Prompts: cost such as Wi-Fi or pay TV/Netflix cost, limited access) |
| ***Strategies to increase PA and reduce screen time***  1) What one strategy do you think would be most effective/important to help increase PA amongst new mothers (i.e. mothers with babies)?  2) What one strategy do you think would be most effective/important to help reduce recreational screen time [enter that screen behaviour here] amongst new mothers (i.e. mothers with babies)? |
